# Synthesis and Biological Evaluation of Thiophene Derivatives as Acetylcholinesterase Inhibitors

**DOI:** 10.3390/molecules17067217

**Published:** 2012-06-12

**Authors:** Mohamed M. Ismail, Mona M. Kamel, Lamia W. Mohamed, Samar I. Faggal, Mai A. Galal

**Affiliations:** 1 Pharmaceutical Organic Chemistry Department, Faculty of Pharmacy, Cairo University, El-Kasr El-Aini Street, Cairo 11562, Egypt; 2 Pharmacology and Toxicology Department, Faculty of Pharmacy, Cairo University, Cairo 11562, Egypt

**Keywords:** thiophene, Gewald, Ellman, acetylcholinesterase, Alzheimer’s disease

## Abstract

A series of new thiophene derivatives has been synthesized using the Gewald protocol. The acetylcholinesterase inhibition activity was assayed according to Ellman’s method using donepezil as reference. Some of the compounds were found to be more potent inhibitors than the reference. 2-(2-(4-(4-Methoxyphenyl)piperazin-1-yl)acetamido)-4,5,6,7-tetrahydrobenzo[*b*]thiophene-3-carboxamide (**IIId**) showed 60% inhibition, compared to only 40% inhibition by donepezil.

## 1. Introduction

Alzheimer’s disease (AD) is a neurodegenerative disorder of the central nervous system (CNS) characterized by premature mental deterioration [1]. Cognitive deficits in AD was found to be associated with disruption of central cholinergic transmission [2]. The successful development of AChE inhibitors was based on the well accepted hypothesis that the decline in cognitive and mental functions associated with AD is related to loss of cortical cholinergic transmission [1].

**Figure 1:**
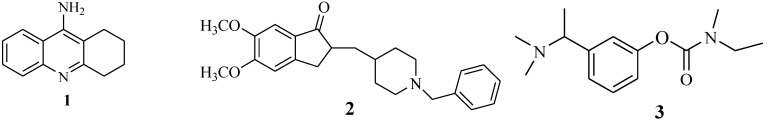
Structures of some acetylcholinesterase inhibitor drugs.

The structural diversity of known AChE inhibitors and the possibility to explore distinct mode of action have stimulated studies that confirmed the activity of new AChE inhibitors such as tacrine (**1**) [3], donepezil (**2**) [4] and rivastigmine (**3**) [5] (Figure 1). Donepezil (E 2020) initiated a new class of AChE inhibitors with longer and more selective action with manageable adverse effects [1]. Recent data has demonstrated that the effect of AChE inhibitors are not necessarily confined to cholinesterase inhibition but that they also improve the symptoms of AD through regulation of the processing and secretion of amyloid precursor protein (APP) [6].

In the present study, and in order to obtain analogues of donepezil that could retain its main spatial and physicochemical characteristics, the indanone moiety of donepezil was replaced by a thiophene ring with a carbonyl group at position 3, an acetamido group was used as a spacer connecting the thiophene ring with arylpiperidines and piperazines [7]. In addition to the focus on the *N*-benzylpiperidines and *N*-benzylpiperazines due to their specificity and potency [8]. Structure elucidation of the newly synthesized derivatives was performed and furthermore, the new derivatives were tested for their capacity to inhibit acetylcholinesterase enzyme.

## 2. Results and Discussion

### 2.1. Chemistry

**Scheme 1:**
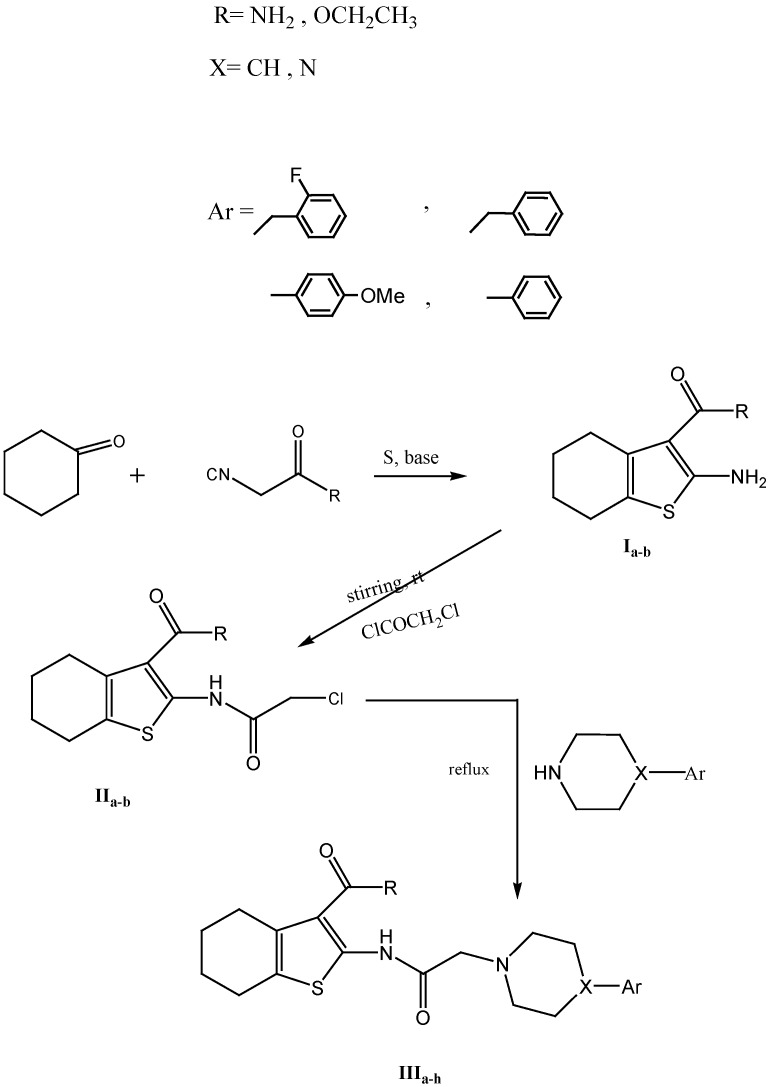
Synthesis of target compounds **IIIa**–**h**.

**Scheme 2:**
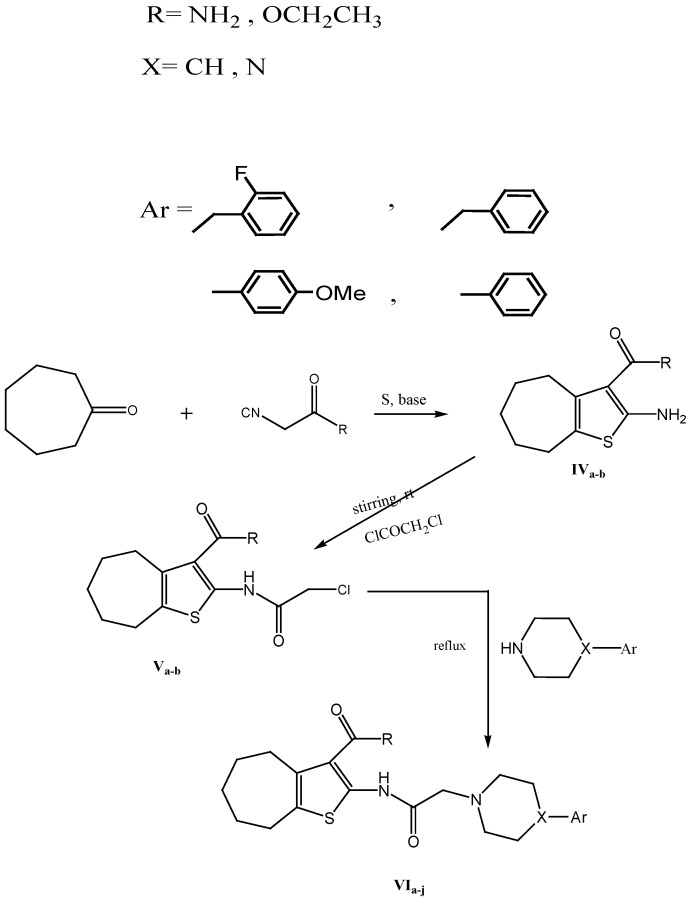
Synthesis of target compounds **VIa**-**j**.

The synthetic pathway for the synthesis of the designed compounds is shown in Scheme 1 and Scheme 2. The starting tetrahydrobenzothiophenes **Ia,b** were synthesized adopting a direct Gewald method, while the synthesis of **IVa** was achieved via a modified direct Gewald procedure. On the other hand, **IVb** was synthesized using Arya`s method, where the reaction proceeded in two steps. The produced **Ia,b** and **IVa,b** was reacted with chloroacetyl chloride by stirring in acetic acid at room temperature to afford **IIa,b** and **Va,b**. The IR spectrum of **Va** showed the appearance of an absorption band at 3,390–3,209 cm^−1^ due to NH and NH_2_ groups together with absorption bands at 1,670 and 1,627 cm^−1^ corresponding to two C=O groups The ^1^H-NMR spectrum showed a singlet signal at δ 4.43 ppm corresponding to CH_2_Cl protons, while the mass spectrum showed the isotopic pattern of chlorine, as well as an exchangeable singlet at δ 11.11 ppm corresponding to the NH protons.

Upon the reaction of **IIa,b and IVa,b** with different amines taking into consideration the desired structure similarity to donepezil, the reaction proceeded for different reflux times adopting several solvent systems while the reactions were monitored using thin layer chromatography.

The structure of the produced final derivatives was confirmed by IR, ^1^H-NMR, ^13^C-NMR, mass spectrometry and microanalyses. The IR spectra showed the appearance of an absorption band in the 3105–3000 cm^−1^ range corresponding to C-H aromatic. Additionally, the ^1^H-NMR showed the appearance of aromatic protons in the range of δ 6.80–7.45 ppm. Moreover, compounds **IIId** and **Vd** showed the appearance of a singlet peak in the range of δ 3.68–4.23 ppm corresponding to the methoxy protons. The ^13^C-NMR of compound **IIIb,d** showed aromatic carbon atoms in the δ 113.05–152.89 ppm range. In addition, the carbon atoms of the piperazine ring appeared in the ^13^C-NMR in a range of δ 49.31–52.47 and 52.80–52.96 ppm, respectively. 

### 2.2. Pharmacology

**Figure 2:**
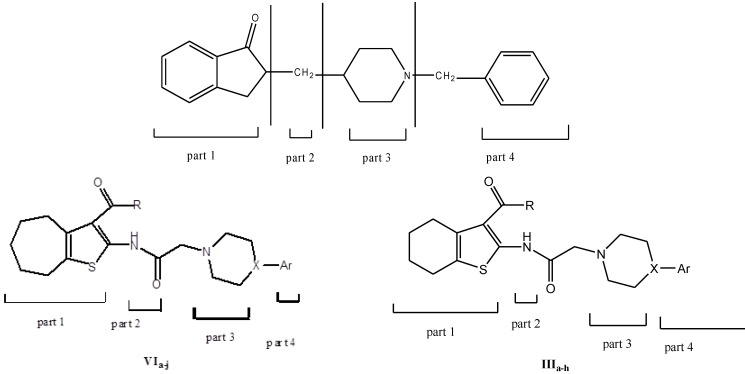
Design strategy of the newly synthesized derivatives.

Donepezil, which is a benzylpiperidine derivative, was chosen as a reference standard drug as it shows potent reversible acetylcholinesterase inhibitor activity [9]. The new derivatives were designed with structural similarity to donepezil where the indanone moiety was replaced by the tetrahydrobenzo[b]- thiophene ring which is expected to act as the peripheral anionic site, while the N atom from the piperazine group acted as the positively charged centre presented in many potent AChE inhibitors and the phenyl group attached to the piperazine acted as the choline binding site [10] (Figure 2)*.*

*In vitro* tests showed that substitution at the *ortho* position of the benzyl ring specifically by a fluorine atom enhances activity [7]. In addition, it was reported that the carbonyl of the acetamido group (COCH_2_) was crucial for activity [11].

On the previous basis, Ellman’s assay method was performed on all of the newly synthesized compounds and on donepezil as standard to measure their inhibitory activity against the enzyme acetylcholinesterase. Some of them such **IIIa**, **IIId**, **VIb**, **VIg** and **VIh** displayed inhibitory activity (56.67, 60, 56.6 and 51.67%, respectively), better than donepezil, which tested under the same conditions showed 40% inhibition. On the other hand, compounds **IIIb** and **IIIf** showed similar activity to donepezil.

### 2.3. Molecular Modelling

The crystal structure of AChE cocrystallized with donepezil shows that donepezil binds in the active site cleft by several interactions through π–π interaction between the indanone moiety and Trp 279 which were considered to be responsible for the observed affinity. In addition there is another π–π interaction between the benzyl ring and Trp 84, and finally, a π- cation interaction between Phe 330 and the charged nitrogen atom of the piperidine ring. No direct hydrogen bond between the enzyme and donepezil could be detected in the structure. Compounds **IIIa**, **IIIb**, **IIIe**, **IIIh**, **VIa**, **VIf** and **VIg** showed good energy scores in addition to their superimposition with donepezil in the active site of AChE. These derivatives formed additional hydrogen bond interactiond with Phe 288 at the AChE active site showing better fitting to the receptor which revealed that these derivatives could have similar or even better activity pharmacological activity than donepezil.

## 3. Experimental

### 3.1. Chemistry

All melting points were determined on a Stuart apparatus and the values given are uncorrected. IR spectra (KBr, cm^−1^) were determined on a Shimadzu IR 435 spectrophotometer (Faculty of Pharmacy, Cairo University, Egypt). ^1^H-NMR and ^13^C-NMR spectra were recorded on Varian Gemini 200 MHz and 300 MHz spectrophotometers (Microanalysis Center, Cairo University, Egypt) using TMS as internal standard. Chemical shift values are recorded in ppm on δ scale. Mass spectra were recorded on a Hewlett Packard 5988 spectrometer (Microanalysis Center, Cairo University, Egypt). Elemental analyses were carried out at the Microanalysis Center, Cairo University, Egypt; found values were within ±0.35% of the theoretical ones. Progress of the reactions was monitored using TLC sheets precoated with UV fluorescent silica gel Merck 60F 254 and were visualized using UV lamp. Compounds **Ia**, **Ib** [12], **IVa ** [13], **IVb ** [14], **IIa**, **IIb** and **Vb ** [15] were synthesized according to reported procedures.

#### 3.1.1. General Procedure for the Synthesis of IIIa–h

To a solution of each of **IIa,b** (0.01 mol) in an appropriate solvent (20 mL), an appropriate amine (0.02 mol) was added and the mixture was heated under reflux for 4–12 h. The product was cooled, the separated solid was filtered, dried and crystallized from ethanol.

*2-(2-(4-Benzylpiperidin-1-yl)acetamido)-4,5,6,7-tetrahydrobenzo[b]thiophene-3-carboxamide* (**IIIa)**. Solvent: ethanol; time of reflux: 12 h; m.p.: 192–194 °C; yield: 80%; IR: 3406, 3290 (NH_2_, NH), 3050 (CH aromatic), 2927, 2912 (CH aliphatic), 1681, 1662 (C=O); ^1^H-NMR (CDCl_3_) δ: 1.62–1.69 (m, 5H, 2CH_2_ + CH piperidine), 1.87 (m, 4H, 2CH_2_), 2.6 (m, 4H, 2CH_2_), 2.74 (m, 4H, 2 CH_2_ piperidine), 3.01 (s, 2H, CH_2_ benzyl), 5.8 (s, 2H, CO-CH_2_-N), 7.22 (s, 2H, NH_2_, D_2_O exchangeable), 7.14–7.31 (m, 5H, aromatic H), 12.63 (s, 1H, NH, D_2_O exchangeable) ppm; MS (*m/z*, % abundance): 411 (M^+^, 8.42%). Anal. calcd. for C_23_H_29_N_3_O_2_S: C 67.12, H 7.10, N 10.21; found C 66.90, H 6.88, N 9.99.

*2-(2-(4-Benzylpiperazin-1-yl)acetamido)-4,5,6,7-tetrahydrobenzo[b]thiophene-3-carboxamide* (**IIIb**). Solvent: ethanol; time of reflux: 12 h; m.p.: 200–202 °C; yield: 80%; IR: 3394, 3209 (NH_2_, NH), 3030 (CH aromatic), 2927, 2850 (CH aliphatic), 1685, 1650 (C=O); ^1^H-NMR (DMSO-d_6_) δ: 1.72 (s, 4H, 2CH_2_), 2.49 (m, 4H, 2CH_2_), 2.6 (m, 4H, 2CH_2_ piperazine), 2.9 (m, 4H, 2CH_2_ piperazine), 3.18(s, 2H, CO-CH_2_-N), 3.47 (s, 2H, CH_2_ benzyl), 7.27 (s, 2H, NH_2_, D_2_O exchangeable), 7.26–7.32 (m, 5H, aromatic protons), 12.27 (s, 1H, NH, D_2_O exchangeable) ppm; ^13^C-NMR (DMSO) δ: 22.41–25.17 (4CH_2_), 52.47 (2CH_2_ piperazine), 52.96 (2CH_2_ piperazine), 60.49 (CO-CH_2_-N), 62.01 (CH_2_ benzyl), 116.23–141.68 (aromatic C), 166.98 (C=O), 167.31 (C=O) ppm; MS (*m/z*, % abundance): 412 (M^+^, 12.31%); Anal. calcd. for C_22_H_28_N4O_2_S: C 64.05, H 6.84, N 13.58; found: C 64.19, H 6.92, N 13.54.

*2-(2-(4-(2-Fluorobenzyl)piperazin-1-yl)acetamido)-4,5,6,7-tetrahydrobenzo[b]thiophene-3-carboxamide* (**IIIc**). Solvent: dioxane; time of reflux: 4 h; m.p.: 196–198 °C; yield: 76%; IR: 3495, 3309 (NH_2_, NH), 3100 (CH aromatic), 2939, 2873 (CH aliphatic), 1675, 1651 (C=O); ^1^H-NMR (DMSO-d_6_) δ: 1.62 (m, 4H, 2CH_2_), 1.87 (m, 4H, 2CH_2_), 2.65–2.75 (m, 8H, 4CH_2_ piperazine), 3.21 (s, 2H, CO-CH_2_-N), 3.63 (s, 2H, CH_2_ benzyl), 7.22 (s, 2H,NH_2_, D_2_O exchangeable), 6.99–7.42 (m, 4H, aromatic H), 12.63 (s, 1H, NH, D_2_O exchangeable) ppm; MS (*m/z*, % abundance): 430 (M^+^, 15.45%); Anal. calcd. for C_22_H_27_FN_4_O_2_S: C 61.37, H 6.32, N 13.01; found: C 61.29, H 6.36, N 13.22.

*2-(2-(4-(4-Methoxyphenyl)piperazin-1-yl)acetamido)-4,5,6,7-tetrahydrobenzo[b]thiophene-3-carboxamide* (**IIId**). Solvent: ethanol; time of reflux: 8 h; m.p.: 234–236 °C; yield: 70%; IR: 3500, 3310 (NH_2_, NH), 3050 (CH aromatic), 2921 (CH aliphatic), 1681, 1651 (C=O); ^1^H-NMR (DMSO-d_6_) δ: 1.72 (m, 4H, 2CH_2_), 2.49–2.5 (m, 4H, 2CH_2_), 2.63–2.65 (m, 4H, CH_2_ piperazine), 3.18 (m, 4H, 2CH_2_ piperazine), 3.3 (s, 2H, CO-CH_2_-N), 3.69 (s, 3H, OCH_3_), 6.88 (s, 2H, NH_2_, D_2_O exchangeable), 6.81–6.91 (dd, 4H, aromatic H), 12.35 (s, 1H, NH ,D_2_O exchangeable) ppm; MS (*m/z*, % abundance): 428 (M^+^, 31.44%); Anal. calcd. for C_22_H_28_N_4_O_3_ S: C 61.66, H 6.59, N 13.07; found C 62.21, H 6.36, N 13.12.

*Ethyl 2-(2-(4-benzylpiperidin-1-yl)acetamido)-4,5,6,7-tetrahydrobenzo[b]thiophene-3-carboxylate* (**IIIe**). Solvent: ethanol; time of reflux: 8 h; m.p.: 80–82 °C; yield: 65%; IR: 3190 (NH), 3028 (CH aromatic), 2931, 2850 (CH aliphatic), 1670 (2C=O); ^1^H-NMR (CDCl_3_) δ: 1.35 (t, 3H, OCH_2_CH_3_), 1.54–1.58 (m, 5H, 2CH_2_ + CH piperidine), 1.71 (m, 4H, 2CH_2_), 2.11 (m, 4H, 2CH_2_), 2.76 (m, 4H, 2CH_2_ piperidine), 3.17 (s, 2H, CH_2_ benzyl), 3.36 (s, 2H, CO-CH_2_-N), 4.3(q, 2H, OCH_2_CH_3_), 7.15–7.30 (m, 5H, aromatic H), 12.63 (s, 1H, NH, D_2_O exchangeable) ppm; MS (*m/z*, % abundance): 440 (M^+^, 23.98%); Anal. calcd. for C_25_H_32_N_2_O_3_: C 68.15, H 7.32, N 6.36; found C 67.55, H6.80, N 6.22.

*Ethyl 2-(2-(4-benzylpiperazin-1-yl)acetamido)-4,5,6,7-tetrahydrobenzo[b]thiophene-3-carboxylate* (**IIIf**). Solvent: Acetonitrile; time of reflux: 12 h; m.p.: 96–98 °C; yield: 60%; IR: 3178 (NH), 3028 (CH aromatic), 2935, 2858 (CH aliphatic), 1670 (2C=O); ^1^H-NMR (CDCl_3_) δ: 1.38–1.42 (t, 3H, OCH_2_CH_3_), 1.79 (m, 4H, 2CH_2_), 2.65 (m, 4H, 2CH_2_), 2.68 (m, 4H, 2CH_2_ piperazine), 2.8 (m, 4H, 2CH_2_ piperazine), 3.3 (s, 2H, CO-CH_2_-N), 3.66 (s, 2H, CH_2_ benzyl), 4.37 (q, 2H, OCH_2_CH_3_), 7.27–7.39 (m, 5H, aromatic H), 12.18 (s,1H, NH, D_2_O exchangeable) ppm; MS (*m/z*, % abundance): 441 (M^+^, 9.77%); Anal. calcd. for C_24_H_31_N_3_O_3_S: C 65.28, H 7.08, N 9.52; found: C 65.32, H7.12, N9.58.

*Ethyl 2-(2-(4-(2-fluorobenzyl)piperazin-1-yl)acetamido)-4,5,6,7-tetrahydrobenzo[b]thiophene-3 carboxylate* (**IIIg**). Solvent: ethanol; time of reflux: 8 h; m.p.: 134–136 °C; yield: 62%; IR: 3450 (NH), 3105 (CH aromatic), 2920 (CH aliphatic), 1674 (2C=O); ^1^H-NMR (DMSO-d_6_) δ: 1.29 (t, 3H, OCH_2_CH_3_), 1.71 (m, 4H, 2CH_2_), 2.5 (m, 4H, 2CH_2_), 2.62 (m, 4H, 2CH_2_ piperazine), 3.06 (m, 4H, 2CH_2_ piprazine), 3.3 (s, 2H, CO-CH_2_-N), 3.62 (s, 2H, CH_2_ benzyl), 4.19 (q, 2H, OCH_2_CH_3_), 7.16–7.45 (m, 4H, aromatic H), 12.02 (s, 1H, NH, D_2_O exchangeable) ppm; MS (*m/z*, % abundance): 459 (M^+^, 0.02%); Anal. calcd. for C_24_H_30_FN_3_O_3_S: C 62.72, H 6.58, N 9.14; found: C 62.79, H6.53, N9.16.

*Ethyl 2-(2-(4-(4-methoxyphenyl)piperazin-1-yl)acetamido)-4,5,6,7-tetrahydrobenzo[b]thiophene-3-carboxylate* (**IIIh**). Solvent: ethanol; time of reflux: 8 h; m.p.: 145–147 °C; yield: 73%; IR: 3240 (NH), 3050 (CH aromatic), 2947, 2904 (CH aliphatic), 1675, 1666 (C=O); ^1^H-NMR (DMSO-d_6_) δ: 1.25–1.3 (t, 3H, OCH_2_CH_3_), 1.72 (m, 4H, 2CH_2_), 2.49–2.6 (m, 4H, 2CH_2_), 2.67 (m, 4H, 2CH_2_, piperazine), 3.13 (m, 4H, 2CH_2_ piperazine), 3.3 (s, 2H, CO-CH_2_-N), 3.7 (s, 3H,OCH_3_), 4.26 (q, 2H, OCH_2_CH_3_), 6.81–6.93 (dd, 4H, aromatic H), 12.04 (s,1H, NH, D_2_O exchangeable) ppm; ^13^C-NMR (DMSO): 13.98 (OCH_2_CH_3_), 22.19–25.71 (4CH_2_), 49.31 (2CH_2_ piperazine), 52.80 (2CH_2_ piperazine), 55.12 (OCH_3_), 59.96 (CO-CH_2_-N), 60.26 (OCH_2_CH_3_), 114.25–152.84 (aromatic C), 164.52 (C=O), 167.68 (C=O) ppm; MS (*m/z*, % abundance): 457 (M^+^, 28.79%); Anal. calcd. for C_24_H_31_N_3_O_4_S; calcd. C 63.00, H 6.83, N 9.18; found: C 63.13, H6.89, N9.21.

*2-(2-Chloroacetamido)-5,6,7,8-tetrahydro-4H-cyclohepta[b]thiophene-3-carboxamide* (**Va**). Chloroacetyl chloride (3.69 g, 0.033 mol) was added to a solution of each of **IVa** (6.3g, 0.03 mol) in acetic acid (20 mL). The reaction mixture was stirred at room temperature for 1 hour. The product was filtered, dried and crystallized from ethanol. M.p.: 220–222 °C; yield 55%; IR: 3390–3209 (NH_2_, NH), 2920 (CH aliphatic), 1670, 1627 (C=O); ^1^H-NMR (DMSO-d_6_) δ: 1.55–1.59 (m, 4H, 2CH_2_), 1.73–1.75 (m, 2H, CH_2_), 2.67–2.71 (m, 4H, 2CH_2_), 4.43 (s, 2H, CO-CH_2_-Cl), 7.5 (s, 2H, NH_2_, D_2_O exchangeable), 11.11 (s, 1H, NH, D_2_O exchangeable) ppm; MS (*m/z*, % abundance): 286 (M^+^, 40.43%), 288 (M + 2, 19.42%); Anal. calcd. for C_12_H_15_ClN_2_O_2_S: C 50.26, H 5.27, N 9.77; found: C 50.29, H5.28, N9.73.

#### 3.1.2. General Procedure of the Preparation of Compounds **VIa**–**j**

To a solution of each of **IVa,b** (0.01 mol) in an appropriate solvent (20 mL), an appropriate amine (0.02 mol) was added and the mixture was heated under reflux for 4–12 h. The product was poured into ice-cold water (25 mL), and then extracted with chloroform. The chloroform layer was dried over anhydrous sodium sulphate. The reaction was filtered and the solvent was evaporated under reduced pressure to give an oily product. The oily product was treated with ether (25 mL) and the solid obtained was filtered, dried and crystallized from ethanol.

*2-(2-(4-Benzylpiperidin-1-yl)acetamido)-5,6,7,8-tetrahydro-4H-cyclohepta[b]thiophene-3-carboxamide* (**VIa**). Solvent: Acetonitrile; time of reflux: 12 h; m.p.: 83–85 °C; yield: 65%; IR: 3325 (NH_2_, NH), 3059, 3024 (CH aromatic), 2920 (CH aliphatic), 1654 (2C=O); ^1^H-NMR (DMSO-d_6_) δ: 1.55–1.58 (m, 4H, 2CH_2_,), 1.78 (m, 2H, CH_2_), 2.49 (m, 4H, 2CH_2_), 2.67–2.74 (m, 5H, 2CH_2_+ CH piperidine), 3.3 (m, 4H, 2CH_2_ piperidine), 3.8 (s, 2H, CH_2_ benzyl), 5.1(s, 2H, CO-CH_2_-N), 7.17–7.29 (m, 5H, aromatic H), 7.45 (s, 2H, NH_2_, D_2_O exchangeable), 10.8 (s, 1H, NH, D_2_O exchangeable) ppm; MS (*m/z*, % abundance): 425.558 (M^+^); Anal. calcd. for C_24_H_31_N_3_O_2_S: C 67.73, H 7.34, N 9.87; found: C 67.48, H7.35, N9.81.

*2-(2-(4-Benzylpiperazin-1-yl)acetamido)-5,6,7,8-tetrahydro-4H-cyclohepta[b]thiophene-3-carboxamide* (**VIb**). Solvent: Acetonitrile; time of reflux: 12; m.p.:108–110 °C; yield: 60%; IR: 3441, 3340 (NH_2_, NH), 3062, 3028 (CH aromatic), 2916 (CH aliphatic), 1651(2C=O); ^1^H-NMR (DMSO-d_6_) δ: 1.52–1.56 (m, 4H, 2CH_2_), 1.78 (m, 2H, CH_2_), 2.49 (m, 4H, 2CH_2_), 2.7 (m, 4H, 2CH_2_ piperazine), 3.13 (m, 4H, 2CH_2_ piperazine), 3.55 (s, 2H, CO-CH_2_-N), 4.09 (s, 2H,CH_2_ benzyl), 7.28–7.32 (m, 5H, aromatic H), 7.45 (s, 2H, NH_2_, D_2_O exchangeable), 11.17 (s, 1H, NH, D_2_O exchangeable) ppm; MS (*m/z*, % abundance): 426.560 (M^+^); Anal. calcd. for C_23_H_30_N_4_O_2_S: C 64.76, H 7.09, N 13.13; found: C 64.82, H 7.11, N13.18.

*2-(2-(4-(2-Fluorobenzyl)piperazin-1-yl)acetamido)-5,6,7,8-tetrahydro-4H-cyclohepta[b]thiophene-3-carboxamide* (**VIc**). Solvent: Dioxane; time of reflux: 4 h; m.p.:170–172 °C; yield: 60%; IR: 3394, 3321 (NH_2_, NH), 3070 (CH aromatic), 2920 (CH aliphatic), 1660–1631 (C=O); ^1^H-NMR(CDCl_3_) δ: 1.52–1.56 (m, 4H, 2CH_2_), 1.78 (m, 2H, CH_2_), 2.49 (m, 4H, 2CH_2_), 2.59 (m, 4H, 2CH_2_ piperazine), 3.05 (m, 4H, 2CH_2_ piperazine), 3.29 (s, 2H, CO-CH_2_-N), 3.6 (s, 2H, CH_2_ benzyl), 7.15–7.44 (m, 4H, aromatic H), 7.22 (s, 2H, NH_2_, D_2_O exchangeable), 8.84 (s, 1H, NH, D_2_O exchangeable) ppm; MS (*m/z*, % abundance): 444.917 (M^+^); Anal. calcd. for C_23_H_29_FN_4_O_2_S: C 62.14, H 6.58, N 12.60; found: C 62.23, H6.56, N12.65.

*2-(2-(4-(4-Methoxyphenyl)piperazin-1-yl)acetamido)-5,6,7,8-tetrahydro-4H-cyclohepta[b]thiophene-3-carboxamide* (**VId**). Solvent: acetonitrile; time of reflux: 12 h; m.p.: 190–192 °C; yield: 60%; IR: 3345, 3440 (NH_2_, NH), 3089 (CH aromatic), 2954 (CH aliphatic), 1778, 1724 (C=O); ^1^H-NMR (DMSO-d_6_) δ: 1.56 (m, 4H, 2CH_2_), 1.8 (m, 2H, CH_2_), 2.49 (m, 4H, 2CH_2_), 2.7 (m, 4H, 2CH_2_ piperazine), 3.16 (m, 4H, 2CH_2_ piperazine), 3.8 (s, 2H, CO-CH_2_-N), 4.23 (s, 3H,OCH_3_), 6.83–7.00 (dd, 4H, aromatic H), 7.48 (s, 2H,NH_2_, D_2_O exchangeable), 11.17 (s, 1H, NH, D_2_O exchangeable) ppm; MS (*m/z*, % abundance): 442.657 (M^+^); Anal. calcd. for C_23_H_30_N_4_O_3_S: C 62.42, H 6.83, N 12.66; found C 62.47, H 6.88, N 12.60.

*2-(2-(4-Phenylpiperazin-1-yl)acetamido)-5,6,7,8-tetrahydro-4H-cyclohepta[b]thiophene-3-carboxamide* (**VIe**). Solvent: acetonitrile; time of reflux: 12 h; m.p.: 158–160 °C; yield: 60%; IR: 3394 (NH_2_, NH), 3050 (CH aromatic), 2920 (CH aliphatic), 1651 (2C=O); ^1^H-NMR (DMSO-d_6_) δ: 1.56 (m, 4H, 2CH_2_), 1.79 (m, 2H, CH_2_), 2.49 (m, 4H, 2CH_2_), 2.7 (m, 4H, 2CH_2_ piperazine), 3.2 (m, 4H, 2CH_2_ piperazine), 3.8 (s, 2H, CO-CH_2_-N), 6.80–7.27 (m, 5H, aromatic H), 7.48 (s, 2H, NH_2_, D_2_O exchangeable), 11.17 (s, 1H, NH, D_2_O exchangeable) ppm; MS (*m/z*, % abundance): 412 (M^+^, 0.75%); Anal. calcd. for C_22_H_28_N_4_O_2_S: C 64.05, H 6.84, N 13.58; found C 63.94, H6.84, N 13.52.

*Ethyl 2-(2-(4-benzylpiperidin-1-yl)acetamido)-5,6,7,8-tetrahydro-4H-cyclohepta[b]thiophene-3-carboxylate* (**VIf**). Solvent: ethanol; time of reflux: 8 h; m.p.: 155–157 °C; yield: 56%; IR: 3332 (NH), 3000 (CH aromatic), 2916 (CH aliphatic), 1716, 1658 (C=O); ^1^H-NMR(CDCl_3_) δ: 1.37–1.42 (t, 3H, OCH_2_CH_3_), 1.59–1.67 (m, 6H, 3CH_2_), 1.83 (m, 4H, 2CH_2_), 2.7 (m, 5H, 2CH_2_ + CH piperidine), 3.03 (m, 4H, 2CH_2_ piperidine), 3.56 (s, 2H, CH_2_ benzyl), 4.33 (s, 2H, CO-CH_2_-N), 4.39 (q, 2H, OCH_2_CH_3_), 7.26–7.27 (m, 5H, aromatic H), 11.78( s,1H, NH, D_2_O exchangeable) ppm; MS (*m/z*, % abundance): 453 (M^+^−H, 1.05%); Anal. calcd. for C_26_H_34_N_2_O_3_S: C 68.69, H 7.54, N 6.16; found C 68.73, H 7.58, N 6.18.

*Ethyl 2-(2-(4-benzylpiperazin-1-yl)acetamido)-5,6,7,8-tetrahydro-4H-cyclohepta[b]thiophene-3-carboxylate* (**VIg**). Solvent: Acetonitrile; time of reflux: 12; m.p.: 153–155 °C; yield: 56%; IR: 3345 (NH), 3020 (CH aromatic), 2920 (CH aliphatic), 1716, 1658 (C=O); ^1^H-NMR (CDCl_3_) δ: 1.37–1.42 (t, 3H, OCH_2_CH_3_), 1.59–1.69 (m, 6H, 3CH_2_), 1.83 (m, 4H, 2CH_2_), 2.7 (m, 4H, 2CH_2_ piperazine), 3.03 (m, 4H, 2CH_2_ piperazine), 3.56 (s, 2H, CO-CH_2_-N), 4.33 (s, 2H, CH_2_ benzyl), 4.39 (q, 2H, OCH_2_CH_3_), 7.26–7.37 (m, 5H, aromatic H), 11.78 (s, 1H, NH, D_2_O exchangeable) ppm; MS: (*m/z*, % abundance) 455 (M^+^, 12.34%); Anal. calcd. for C_25_H_33_N_3_O_3_S: C 65.90, H 7.30, N 9.22; found C 65.94, H 7.31, N 9.29.

*Ethyl 2-(2-(4-(2-fluorobenzyl)piperazin-1-yl)acetamido)-5,6,7,8-tetrahydro-4H-cyclohepta [b]thiophene-3-carboxylate* (**VIh**). Solvent: Ethanol; time of reflux: 8 h; m.p.: 163–165 °C; yield: 55%; IR: 3345 (NH), 3020 (CH aromatic), 2920 (CH aliphatic), 1716, 1658 (C=O); ^1^H-NMR (CDCl_3_) δ: 1.37–1.42 (t, 3H, OCH_2_CH_3_), 1.57–1.67 (m, 6H, 3CH_2_), 1.85 (m, 4H, 2CH_2_), 2.71 (m, 4H, 2CH_2_ piperazine), 3.03 (m, 4H, 2CH_2_ piperazine), 3.56 (s, 2H, CO-CH_2_-N), 4.33 (s, 2H, CH_2_ benzyl), 4.39 (q, 2H, OCH_2_CH_3_), 7.16–7.45 (m, 4H, aromatic H), 11.78 (s, 1H, NH, D_2_O exchangeable) ppm; MS (*m/z*, % abundance): 473 (M^+^, 100%); Anal. calcd. for C_25_H_32_FN_3_O_3_S: C 63.40, H 6.81, N 8.87; found: C 63.49, H 6.84, N 8.83.

*Ethyl 2-(2-(4-(4-methoxyphenyl)piperazin-1-yl)acetamido)-5,6,7,8-tetrahydro-4H-cyclohepta[b]thiophene-3-carboxylate* (**VIi**). Solvent: Ethanol; time of reflux: 8; m.p.: 128–130 °C; yield: 60%; IR: 3259 (NH), 3020 (CH aromatic), 2924, 2850 (CH aliphatic), 1683 (C=O); ^1^H-NMR (DMSO-d_6_) δ :1.26–1.33 (t, 3H, OCH_2_CH_3_), 1.54 (m, 4H, 2CH_2_), 1.8 (m, 2H, CH_2_), 2.5 (m,4H, 2CH_2_), 2.66 (m, 4H, 2CH_2_ piperazine), 3.0 (m, 4H, 2CH_2_ piperazine), 3.3 (s, 2H, CO-CH_2_-N), 3.68 (s, 3H, OCH_3_), 4.27 (q, 2H, OCH_2_CH_3_), 6.81–6.92 (dd, 4H, aromatic H), 11.8(s,1H, NH, D_2_O exchangeable) ppm; ^13^C-NMR (DMSO-d_6_) δ: 14.13 (OCH_2_CH_3_), 26.54–31.54 (5CH_2_), 49.42 (2CH_2_ piperazine), 52.89 (2CH_2_ piperazine), 55.12 (OCH_3_), 60.36 (CO-CH_2_-N + OCH_2_CH_3_), 113.05–152.89 (aromatic C), 164.58 (C=O), 167.73 (C=O) ppm; MS (*m/z*, % abundance): 471 (M^+^, 28.99%); Anal. calcd. for C_25_H_33_N_3_O_4_S: C 63.67, H 7.05, N 8.91; found C 63.6, H 7.05, N 8.90.

*Ethyl 2-(2-(4-phenylpiperazin-1-yl)acetamido)-5,6,7,8-tetrahydro-4H-cyclohepta[b]thiophene-3-carbox-ylate* (**VIj**). Solvent: Ethanol; time of reflux: 8 h; m.p.: 138–140 °C; yield: 65%;IR: 3332 (NH), 3100 (CH aromatic), 2920 (CH aliphatic), 1716, 1658 (C=O); ^1^H-NMR (DMSO-d_6_) δ: 1.29–1.30 (t, 3H, OCH_2_CH_3_), 1.32 (m, 2H, CH_2_), 1.54 (m, 4H, 2CH_2_), 1.8 (m, 2H, CH_2_), 2.71 (m, 4H, 2CH_2_ piperazine), 2.98 (m, 2H, CH_2_), 3.32 (m, 4H, CH_2_ piperazine), 3.3 (s, 2H, CO-CH_2_-N), 4.26 (q, 2H, OCH_2_CH_3_), 6.93–7.24 (m, 5H, aromatic H), 11.83 (s, 1H, NH, D_2_O exchangeable) ppm. MS (*m/z*, % abundance): 441 (M^+^, 17.79%); Anal. calcd. for C_24_H_31_N_3_O_3_S: C 65.28, H 7.08, N 9.52; found C 65.26, H 7.09, N 9.47.

### 3.2. Pharmacology

All of the newly synthesized compounds were subjected to an AChE inhibitory activity test. Donepezil, which is a benzylpiperidine derivative, was chosen as a reference standard drug as it shows potent anticholinesterase activity. Adult male albino Wister rats weighing 180–200 g were used in the present study. Rats were purchased from the animal house of El-Nile Company (Cairo, Egypt). Rats were kept under constant laboratory conditions and were allowed free access to food and water throughout the period of investigation. The tested compounds were orally administered at concentration of 2.6351 mM (equivalent to that of donepezil). The compounds were mixed with Tween 80, diluted with distilled water and administered orally. After 30 minutes rats were killed, decapitated, then brains were carefully removed and homogenized in normal saline (pH 7.4).

Inhibitory activity against AChE was evaluated at 37 °C by the colorimetric method reported by Ellman *et al.* [16]. The principle of the assay is based on that the thio-ester substrate acetylthiocholine (AchSC) is hydrolyzed by the enzyme, releasing a sulfhydrylic group able to react with bis (3-carboxy-4-nitrophenyl) disulfide (Ellman’s reagent). The kinetics of this activity is then followed with the use of a spectrophotometer at 412 nm for 2 min. Absorbance is measured at 0, 1 and 2 min and the mean change in absorbance (ΔA) is calculated for each sample the values were recorded. The AChE inhibition was determined for each compound. Each assay was run in triplicate and each reaction was repeated three independent times (Table 1).

**Table 1:** Inhibition of AChE activity of donepezil and the synthesized anticholinesterase compounds.

Compound Number	Choline Esterase Content (U/gm Wet Weight)	% Inhibition
Normal saline	2815.20 ± 171.33	0%
donepezil	1689.20 ± 172.42	40%
III_a_	1219.20 ± 87.78	56.67%
III_b_	1689.12 ± 136.79	40%
III_c_	2404.65± 200.33	14.58%
III_d_	1126.08 ± 87.78	60%
III_e_	1853.24 ± 183.18	34.17%
III_f_	1736.04 ± 119.62	38.33%
III_g_	22287.70 ± 400.60	20.83%
III_h_	1829.88 ± 136.79	35%
VI_a_	2111.40 ± 209.83	25%
VI_b_	1360.68 ± 114.93	51.67%
VI_c_	1876.80 ± 82.94	33.33%
VI_d_	1906.12 ± 168.46	32.29%
VI_e_	2017.56 ± 93.84	28.33%
IV_f_	1876.68 ± 104.92	33.34%
IV_g_	1219.20 ± 87.78	56.67%
IV_h_	1360.56 ± 87.76	51.67%
IV_i_	2533.68 ± 201.81	10%
IV_j_	2627.52 ± 46.92	6.67%

### 3.3. Molecular Modelling

**Figure 3:**
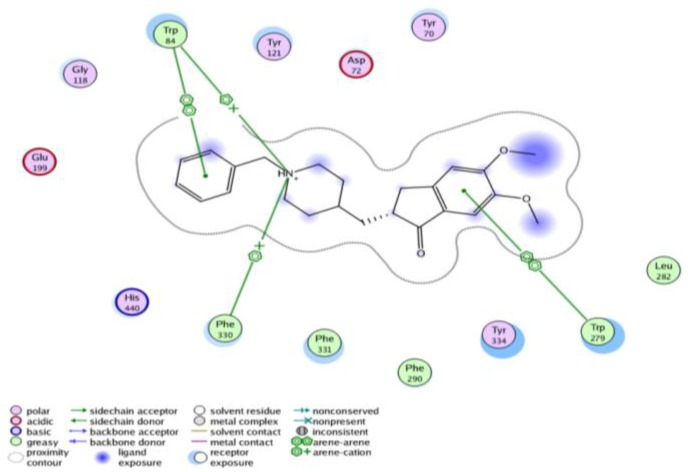
Interactions of donepezil with the amino acids of the active site of AChE.

**Figure 4:**
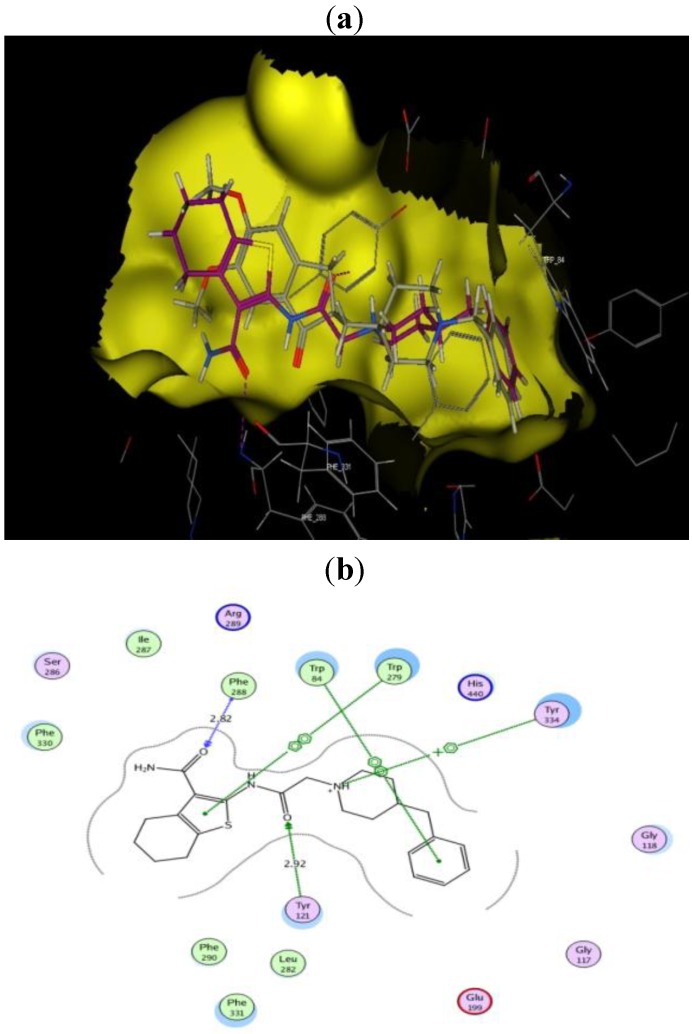
(**a**) Docked conformation alignment of **III**_a_ (red) and its original co-crystallized ligand (grey) in the AChE binding site generated by MOE docking. (**b**) simplified structure showing interaction between **III**_a_ and the aromatic residues in the AChE active site.

**Figure 5:**
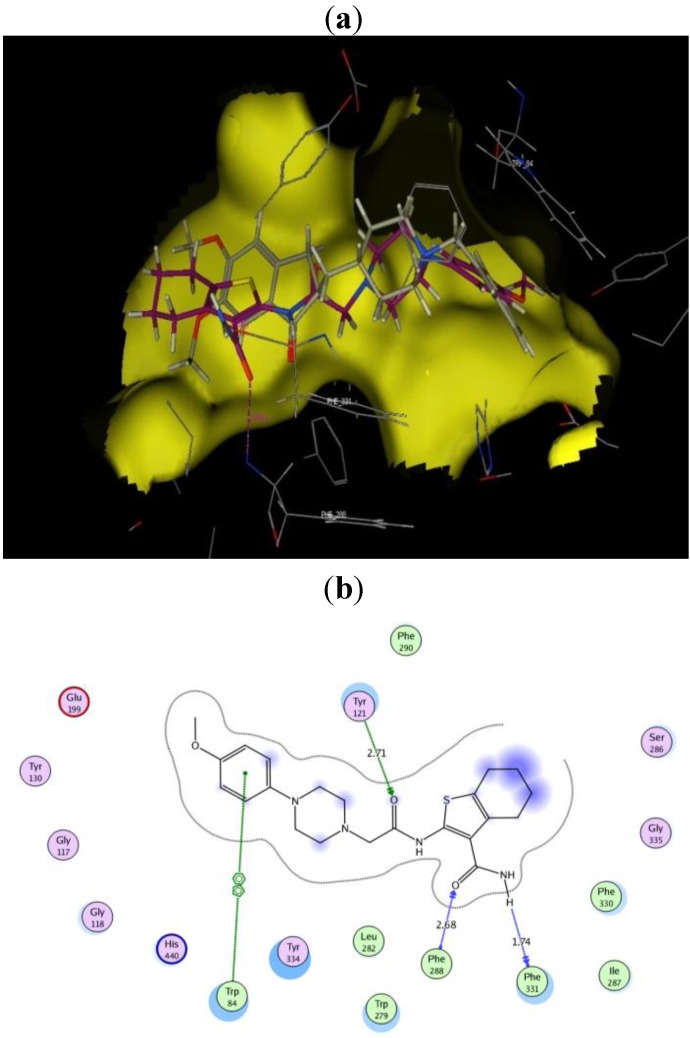
(**a**) Docked conformation alignment of **III**_d_ (red) and its original co-crystallized ligand (grey) in the AChE binding site generated by MOE docking. (**b**) simplified structure showing interaction between **III**_d_ and the aromatic residues in the AChE active site.

Docking was carried out on an Intel Pentium 1.6 GHz processor, 512 MB memory with Windows XP operating system using Molecular Operating Environment (MOE 2008.10; Chemical Computing Group, Montereal, Canada) as the computational software. The 3D structure of the acetylcholine esterase complexed with donepezil was obtained from the Protein Data Bank (PDB ID: 1EVE) at Research Collaboration for Structural Bioinformatics (RCSB) protein data bank base [17] with a 2.5 A resolution. In the present work, all the prepared new compounds were docked using a rigid receptor/ fexible ligand approach adopting five energy maps which are hydrophobicity, electrostatic, hydrogen bond formation and two Van der Waal parameters. The docking scores were expressed in negative energy terms; the lower the binding free energy, the better the binding affinity. The data obtained from docking of the target compounds were explained in Table 2, Figure 3, Figure 4 and Figure 5.

**Table 2:** MOE Scores of Donepezil, compounds III_a–h_ and VI_a–j_, and bonds formed with amino acid residues and their lengths.

Compound Number	Type of Interaction (Amino Acid Residues, Length of Bond in A)	Binding Energy Score (Kcal/mol)
Donepezil	π-π (Trp279), π-π, π-cation (Trp84), π-cation (Phe330)	−31.1758
III_a_	π-π (Trp279), π-π (Trp84), H-bond (Tyr121, 2.92), π-cation (Trp334), H-bond (Phe288, 2.82)	−29.5362
III_b_	π-π (Trp84), π-cation (Trp334), π-cation (Phe330), H-bond (Phe331, 2.00)	−29.2693
III_c_	π-π (Trp279), π-cation (Tyr334), π-cation (His440)	−25.7001
III_d_	π-π (Trp84), H-bond (Tyr121, 2.7), H-bond (Phe331, 1.74), H-bond (Phe288, 2.68)	−22.5618
III_e_	π-π (Trp279), π-π (Trp84), π-cation (Tyr334)	−28.4632
III_f_	π-π (Trp279), π-π (Trp84), π-cation (Tyr334)	−27.6379
III_g_	π-π(Trp279)	−20.3797
III_h_	π-π (Trp279), π-π (Trp84)	−28.0285
VI_a_	π-π (Trp279), π-π(Trp84), H-bond (Tyr70, 1.94)	−27.0547
VI_b_	π-π, π-cation (Trp84), π-cation (Phe330)	−26.6091
VI_c_	π-π (Trp279), π-π, π-cation (Trp84), π-cation (Phe330)	−26.2333
VI_d_	π-π (Trp279), π-π (Trp84)	−21.6314
VI_e_	π-π (Trp84), H-bond (Tyr121, 2.58)	−23.8446
VI_f_	π-π (Trp279), π-π (Trp84), π-cation (Tyr334), H-bond (Tyr121, 2.98)	−29.9461
VI_g_	π-π (Trp279), π-π (Trp84), π-cation (Phe330)	−30.4078
VI_h_	π-π (Trp279), π-π (Trp84), π-cation (Phe330)	−26.7772
VI_i_	H-bond (Tyr121, 2.84), H-bond (Gly119, 2.86), H-bond (ser200, 2.51)	−21.0334
VI_j_	H-bond (Tyr121, 2.74), π-π (Trp84)	−23.3871

## 4. Conclusions

Compound **IIId** showed better inhibitory activity than donepezil owing mainly to its amide group carbonyl that leads to extra binding to the receptor by an H-bond with Phe288, also due to the fact that this particular derivative can bind to the receptor with three different H-bonds leading to better fitting to the receptor. Compounds **VIb**, **VIg** and **VIh** bearing benzylpiperazine and 2-fluorobenzyl piperazine groups with no H-bonding to the receptor showed less inhibitory activity than **IIId** but still better activity than donepezil. Compounds **IIIb and IIIf** having a benzylpiperazine group showed moderate activity, but still retained similar inhibitory activity to donepezil owing to the fitting to the receptor with an extra H-bond of **IIIb** and the π-cation interaction of both compounds**.** From the previous results, the extra binding to the receptor with the H-bond lead to better pharmacological activity.
